# Antifungal Drug Plasma Exposures: A Possible Contribution of Vitamin D-Related Gene Variants

**DOI:** 10.3390/ph15050630

**Published:** 2022-05-20

**Authors:** Jessica Cusato, Alice Palermiti, Alessandra Manca, Jacopo Mula, Miriam Antonucci, Amedeo De Nicolò, Sarah Allegra, Silvia De Francia, Francesco Chiara, Giovanni Di Perri, Francesco Giuseppe De Rosa, Andrea Calcagno, Antonio D’Avolio

**Affiliations:** 1 Laboratory of Clinical Pharmacology and Pharmacogenetics, Department of Medical Sciences, University of Turin, Amedeo di Savoia Hospital, 10149 Turin, Italy; jessica.cusato@unito.it (J.C.); alice.palermiti@unito.it (A.P.); amedeo.denicolo@unito.it (A.D.N.); antonio.davolio@unito.it (A.D.); 2 ASL Città di Torino, Amedeo di Savoia Hospital, 10149 Turin, Italy; miriam.antonucci20@gmail.com; 3 Department of Clinical and Biological Sciences, University of Turin, San Luigi Gonzaga Hospital, Regione Gonzole 10, Orbassano, 10043 Turin, Italy; sarah.allegra@unito.it (S.A.); silvia.defrancia@unito.it (S.D.F.); 336124@edu.unito.it (F.C.); 4 Unit of Infectious Diseases, Department of Medical Sciences, University of Turin, Amedeo di Savoia Hospital, 10149 Turin, Italy; giovanni.diperri@unito.it (G.D.P.); andrea.calcagno@unito.it (A.C.); 5 Unit of Infectious Diseases, Department of Medical Sciences, University of Turin, City of Health and Sciences, 10126 Turin, Italy; francescogiuseppe.derosa@unito.it

**Keywords:** genetics, azoles, pharmacokinetics, *VDR*, *CYP27B1*, *CYP24A1*, *GC*

## Abstract

Vitamin D (VD) seems to influence drug clearance and outcome. Antifungal drugs (AFU) are the most used azoles in clinical practice. In the literature, no data are available concerning VD’s impact on AFU therapy. The aim of this study was to analyze if VD pathway-related polymorphisms may influence voriconazole (VRC), itraconazole (ITC), and posaconazole (PSC) drug concentrations in order to identify patients with the highest probability of response and toxicity. Allelic discrimination was performed through real-time PCR, whereas drug concentrations were through liquid chromatography. A total of 636 samples of AFU-treated patients were included in the analysis. Concerning VRC, concentrations higher than the 1000 ng/mL efficacy cut-off value were predicted by Caucasian ethnicity, *CYP24A1* 3999, and *CYP27B1* + 2838 polymorphisms, whereas levels higher than the 5000 ng/mL toxicity value by Caucasian, female sex, e.v. administration, and *GC* 1296. Considering PSC, concentrations higher than the 700 ng/mL efficacy cut-off value were predicted by *VDR* Cdx2, *CYP27B1* − 1260, and *GC* 1296. Finally, for ITC, *VDR* BsmI was the only predictor of drug exposure higher than the 500 ng/mL efficacy cut-off value, whereas female sex, *CYP27B1* − 1260, and *VDR* TaqI remained in the final regression model related to concentrations higher than the 1000 ng/mL toxicity-associated cut-off value. This is the first study reporting the influence of VD pathway-related gene SNPs on AFU exposures, efficacy, and toxicity.

## 1. Introduction

Azoles are the largest class of antifungal drugs (AFU) for clinical use. The first generation of azole drugs includes miconazole, whereas itraconazole (ITC) belongs to the second generation, and it is available in both endovenous (e.v.) and oral (p.o.) formulations. Following further discoveries, the third-generation broad-spectrum azoles were introduced: voriconazole (VRC), posaconazole (PSC), and isavuconazole. The merits of these recent azoles are substantial, as they have improved antifungal activity, safety, and pharmacokinetics (PK) [1]. Azoles reduce ergosterol synthesis by inhibition of lanosterol 14α-demethylase, a CYP450-dependent enzyme. The ergosterol is replaced by unusual methylated sterols, and the normal permeability and fluidity of the fungal membrane are compromised; thus, the mycete dies [2].

Several studies are present in the literature concerning AFU clinical trial data, but their optimal clinical use is still debated [3]: the combination of PK, pharmacodynamics (PD), and pharmacogenetics (PG) could be an important tool in order to personalize treatment, identifying a range of strategies that should be important for AFU optimal clinical use [4]. As an example, a PK/PD model for VRC could be useful in preventing invasive pulmonary aspergillosis, providing a prophylactic AFU regimen: in a study by Taotao Wang et al. based on a Monte Carlo simulation, authors suggested 200 mg of VRC e.v. or p.o. twice daily for Aspergillus infections and 300 mg administered p.o. twice daily or with 200 mg administered e.v. twice daily for Candida infections [5].

It is known that the vitamin D (VD) pathway may have an impact on drug clearance and therapy outcome: in this context, Lindh et al. showed that seasonal variation in VD plasma levels (due to low or high light exposure) was associated with immunosuppressant drug concentrations. The two analyzed drugs (sirolimus and tacrolimus) are metabolized by CYP3A4 and showed variations during the year according to VD levels; supporting this, the enzyme induction increases when VD levels are high, leading to reduced drug exposure [6]. Other factors could impact seasonality: for example, the nitric oxide is released in tissue after solar exposition, leading to a darker skin pigmentation [7].

Recently, our group described anti-HIV drug plasma variation over a period of 10 years: we suggested an annual fluctuation in samples for lopinavir, etravirine, and maraviroc. In detail, lopinavir showed higher concentrations in winter than in summer, whereas the other drugs had an opposite trend [8]. Moreover, we found that seasonality influenced the achievement of etravirine efficacy concentrations of 300 ng/mL. We concluded that VD genetics has to be investigated in order to understand the possible mechanism underlying these seasonal drug variations.

VD modulates the enzymes involved in drug metabolism and elimination through a classic activation of VD receptor (VDR)-mediated gene transcription [9].

Seasonal AFU fluctuations data are not available since these treatments are generally used for a brief period of time: for this reason, the aim of this study was to analyze if polymorphisms related to this pro-hormone pathway may influence VRC, ITC, and PSC drug concentrations. This could lead to the identification of VD-related predictors useful for selecting patients with the highest probability of drug response and those more predisposed to toxicity.

## 2. Results

In this retrospective study, 636 samples of patients treated with AFU were included in the analysis.

### 2.1. Voriconazole

Patient characteristics are reported in Table 1: 357 subjects administered with VRC were analyzed; their median age was 51 years, females were 235, and the medium BMI was 21.9 kg/m^2^.

**Figure 1:**
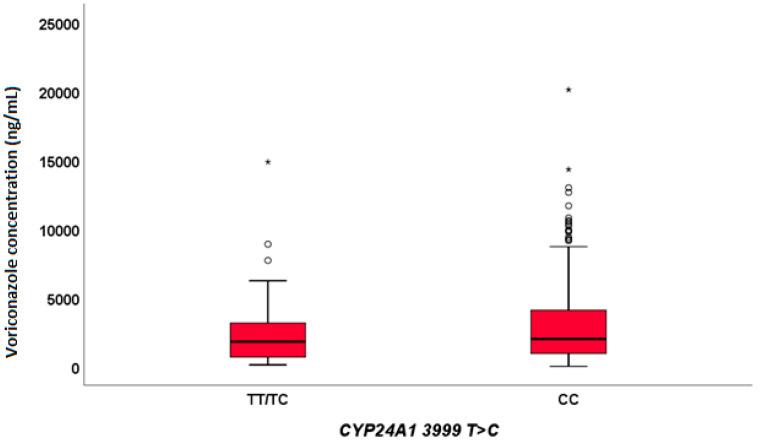
Influence of *CYP24A1* 3999 T > C SNP on voriconazole exposure (*p* = 0.007). Outliers are represented by little circles, and extreme outliers are represented by little stars.

A statistically significant positive correlation between age and VRC exposure was observed (*p* = 0.015, PC = 0.129). Furthermore, *CYP24A1* 3999 CC was found to influence VRC levels, both in the total population (*p* = 0.007, Figure 1) and in 184 individuals who were treated with the most frequent VRC posology of 200 mg (bid in day) BID (*p* = 0.034).

Finally, logistic regression analyses evaluating predictors of concentration higher than the efficacy cut-off value of 1000 ng/mL and the toxicity limit of 5000 ng/mL were performed; concerning the first target (Table 2), Caucasian ethnicity, *CYP24A1* 3999 CC, and *CYP27B1* + 2838 TT genotypes were predictive parameters, whereas Caucasian, female sex, e.v. administration and *GC* 1296 AC/CC genotype patients were for the second aim (Table 2).

### 2.2. Posaconazole

Characteristics of individuals treated with PSC are reported in Table 1: for 136 subjects, the median age was 51 years, females were 66, and the medium BMI was 24.2 kg/m^2^.

No correlation between age, BMI, creatin levels, and PSC exposure was suggested.

**Figure 2:**
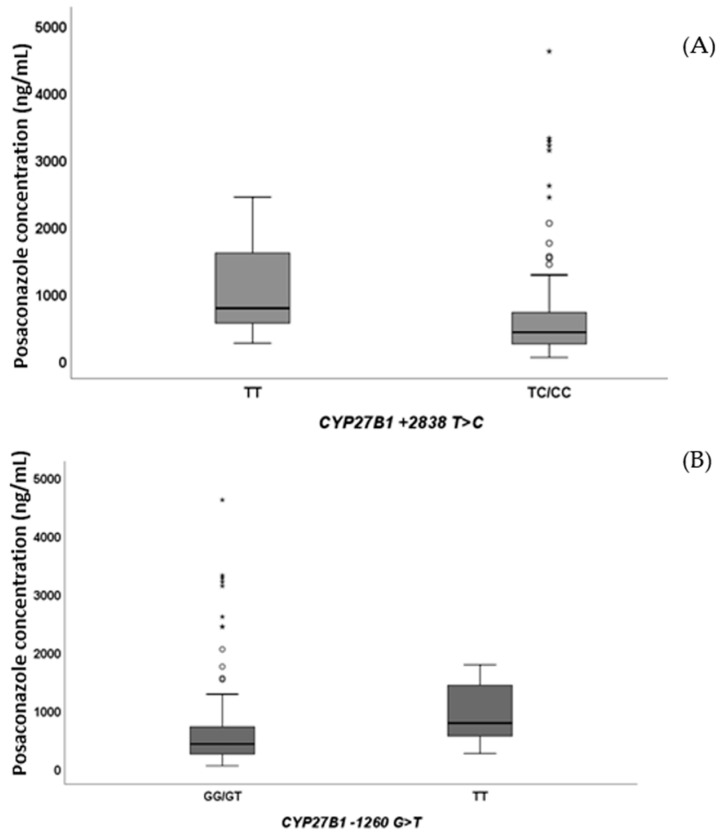
*CYP27B1* + 2838 T > C ((**A**), *p* = 0.038) and *CYP27B1* − 1260 G > T ((**B**), *p* = 0.004) SNPs influence on posaconazole exposure. Outliers are represented by little circles, and extreme outliers are represented by little stars.

Considering the genetic analyses, *CYP27B1* + 2838 TC/CC (*p* = 0.038, Figure 2A) and *CYP27B1* − 1260 TT (*p* = 0.004, Figure 2B) were able to affect PSC plasma exposure.

**Figure 3:**
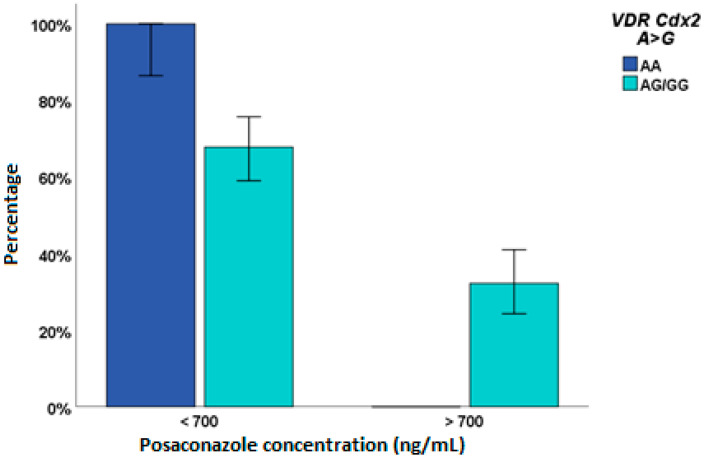
Influence of VDR Cdx2 A > G SNP on posaconazole exposure.

Only one patient showed a PSC concentration higher than the toxicity cut-off value of 3500 ng/mL. For this reason, only the efficacy (levels > 700 ng/mL) logistic regression analysis was performed (Table 3), and *VDR* Cdx2 AG/GG (Figure 3), *CYP27B1* − 1260 TT, and *GC* 1296 CC genotypes were predictors.

### 2.3. Itraconazole

A total of 143 pediatric (median age = 9 years) patients were analyzed: their characteristics are resumed in Table 1. Females were 87, and the medium BMI was 16.5 kg/m^2^.

Concerning age, BMI, creatin levels, and ITC plasma concentrations, no correlations were highlighted.

**Figure 4:**
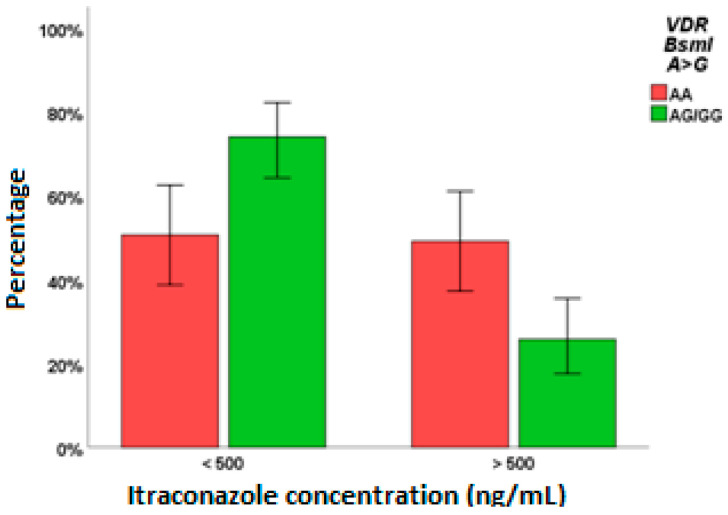
Influence of *VDR* BsmI A>G SNP on ITC exposure. *p =* 0.002.

*VDR* BsmI AG/GG (*p* = 0.002, Figure 4), *VDR* ApaI AA (*p* = 0.035), and *CYP24A1* 8620 AG/GG (*p* = 0.020) affected ITC levels.

In the regression analysis evaluating concentration higher than the efficacy cut-off value of 500 ng/mL (Table 4A), *VDR* BsmI AG/GG was the only predictor. On the other hand, the female sex, *CYP27B1* − 1260 GT/TT and *VDR* TaqI CC remained in the final regression model related to the toxicity-associated cut-off value of 1000 ng/mL (Table 4B).

## 3. Discussion

Azoles are responsible for PK interactions: AFU are both substrates and inhibitors, in particular of CYP2C9 and CYP3A4, which metabolize some drugs; this could lead to increased blood and tissue exposures with possible side effects [1]. For these reasons, it is important to monitor and predict AFU concentrations, also according to genetics [10].

Our group explored the role of some SNPs of encoding enzymes and transporters associated with AFU metabolism and elimination: VRC plasma levels were affected by *SLCO1B3* c.334, ABCG2 c.1194 + 928 CC, and *ABCC2* c. − 24; in addition, sex, ethnicity, *SLCO1B3* c.334 GT/TT, and *ABCB1* c.3435 TT remained in the final regression model as VRC plasma exposure predictive factors [11].

VD has a role in affecting the immune-modulation, anti-inflammatory, and antimicrobial activities, but also drug exposures [6,8,12,13]. In fact, Lindh et al. observed high VD levels associated with extended enzymes and transporters induction: this leads to increased drug metabolism and elimination, thus reduced drug exposures. No data are present in the literature concerning AFU treatment and VD-related gene SNPs: for this reason, the aim of this study was to investigate their role in this context.

In this study, Caucasian ethnicity, *CYP24A1* 3999 CC, and *CYP27B1* + 2838 TT genotypes were predictors of concentrations higher than the efficacy cut-off value in 357 VRC-treated patients.

*CYP24A1* 3999 C allele was associated with increased CYP24A1 mRNA expression, consequently decreased VD levels, and, possibly, the reduced probability of having efficacy [12]. In contrast, in our work, we found CC genotype related to VRC exposure higher than 1000 ng/mL, but this could be due to lower VD levels leading to a longer VRC permanence in blood [8].

*CYP27B1* + 2838 CC genotype leads to reduced calcitriol synthesis, causing a lower response since the activated VD hormonal form has an impact on innate and adaptative immune pathways [14]. These data support our findings showing TT genotype as a positive predictor of VRC efficacy.

Specifically, *CYP27B1* − 1260 is in complete linkage disequilibrium with *CYP27B1* + 2838: the GT/TT genotype was a positive predictive factor of PSC concentrations > 700 ng/mL, whereas the TT genotype was negative for ITC toxicity cut-off value. Since the *CYP27B1* − 1260 TT genotype, as *CYP27B1* + 2838 CC, is associated with reduced VD synthesis, we could expect reduced active VD, thus decreased protective function and higher drug exposures. We found this variant related to the PSC efficacy cut-off level, with a negative prediction for the ITC toxicity-related limit; thus, our data seem to be in contrast compared to the literature, and they have to be confirmed in other and larger studies.

Caucasian ethnicity remained in the final regression analyses for both VRC efficacy and toxicity as a positive predictive factor, probably due to other genetic characteristics or parameters affecting drug absorption, distribution, metabolism, and elimination. E.v. administration is a predictor of VRC concentration higher than the toxicity cut-off value, probably due to the increased levels reached by the e.v. dose compared to the p.o. administration. In this context, also *GC* 1296 AC/CC was a positive predictor of this cut-off: the literature suggests that VD levels are not influenced by this variant [15,16]. A study highlighted the *GC* 1296/1307 diplotype (the two variants are in complete linkage disequilibrium) associated with a more likely altered transcription rate, changes in mRNA stability, or a self-clearance of the protein affecting VDBP serum concentrations [15]. Considering these data, our results seem to be the opposite since TG/GG genotype could be associated with higher VDBP levels; thus, higher VD transport to its targets in order to perform its activities (reducing drug concentration and/or modulating protective functions); the same SNP was associated with PSC efficacy cut-off as a negative predictor and, also, in this case, results did not agree with what expected.

*VDR* gene variants were found associated with both PSC and ITC efficacies and ITC toxicity: Cdx2 AG/GG for PSC > 700 ng/mL, BsmI AG/GG for ITC > 500 ng/mL and TaqI CC for > 1000 ng/mL. Particularly, all the AA genotype patients for *VDR* Cdx2 had PSC levels lower than 700 ng/mL: this genotype was associated with higher *VDR* transcriptional activity compared to the GG genotype, thus probably higher *VDR* expression and activity, leading to a possible decrease in PSC metabolism. BsmI GA/AA was highlighted as a predictor of ITC > 500 ng/mL; data in the literature already showed AA genotype associated with lower VD levels, according to our findings [17].

TaqI TC/CC was a predictor of ITC concentration higher than 1000 ng/mL: in 2013, Dongmei Yu et al. in 2013 showed CC genotype associated with lower VD levels according to our results, suggesting an increased ITC exposure related to toxicity [18]. The same was described for female patients, who showed an increased risk of having a concentration higher than the toxicity cut-off value since they have a smaller volume of distribution [19].

Finally, the only suggested correlation was between age and VRC exposure: higher drug plasma levels are more frequent in older patients. These data confirm what is shown in the literature: Cheng et al. suggested a median VRC trough concentration of 4.31 μg/mL for elderly patients compared to the 3.11 μg/mL for adult patients [20].

Some limitations are present in this study: concerning ITC, only pediatric patients were investigated; thus, we have to consider that their capability in metabolizing drugs is different compared to adults; consequently, further studies have to focus on adults in order to highlight differences.

In addition, VD levels were not quantified: this could be a problem since we do not have an overall picture. In fact, we could suppose VD SNPs have an impact, considering genetic predisposition, but we have to understand if different VD metabolites could affect the clinical outcome according to the seasonality, the supplementation, or the patient’s sun exposure.

Finally, different and larger cohorts are needed to be analyzed to reinforce these data.

## 4. Materials and Methods

### 4.1. Characteristics of Enrolled Patients

Samples were obtained from two centers in Piedmont (Italy): the Laboratory of Clinical Pharmacology and Pharmacogenetics (Department of Medical Sciences, University of Turin, ASL “Città di Torino”, Turin, Italy) and Clinical Pharmacology Service “Franco Ghezzo” (Department of Biological and Clinical Sciences, University of Turin, S. Luigi Gonzaga Hospital).

**Figure 5:**
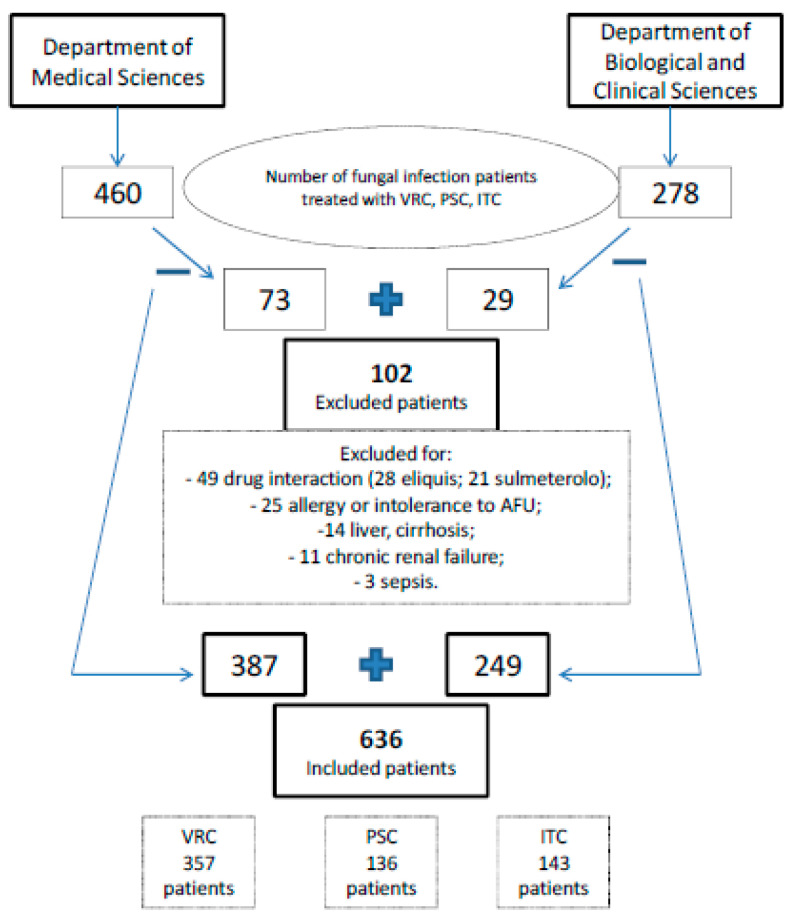
Diagram of patients enrolment considering inclusion and exclusion criteria.

Inclusion criteria were: invasive fungal infection diagnosis and VRC-PSC or ITC treatment, for prophylaxis or therapy purposes, with a compliance of 90%. Individuals with potential interacting drugs (such as some anti-HIV, immunosuppressant, rifampin, rifabutin, carbamazepine, and long-acting barbiturates drugs), allergy or intolerance to AFU, liver cirrhosis, severe malnutrition, chronic renal failure (with estimated creatinine clearance, eCRCl < 60 mL min^−1^) or sepsis diagnosis were excluded (Figure 5).

The study protocol was approved by the local ethics committee (PkPG_J02AC “Studio retrospettivo per la valutazione farmacocinetica e farmacogenetica della terapia antimicotica con farmaci triazolici”). Written informed consent for the study was obtained from each patient, signed by the natural/biological father or mother of a child with full parental legal rights.

### 4.2. Pharmacogenetic Analyses

Patients’ DNA was extracted from whole blood samples with a semi-automated instrument (MagnaPure Compact, Roche, Monza, Italy). Genotypes were assessed with allelic discrimination (Taqman) through a real-time polymerase chain reaction allelic discrimination system (LightCycler 96, Roche, Monza, Italy).

The following gene SNPs were investigated:

*CYP27B1*: rs4646536 (+2838) C > T, rs10877012 (−1260) G > T;

*CYP24A1*: rs927650 C > T, rs2248359 T > C, rs2585428 A > G;

*VDR*: rs731236 (TaqI) T > C, rs10735810 (FokI) T > C, rs11568820 (Cdx2) A > G, rs1544410 (BsmI) G > A, rs7975232 (ApaI) C > A;

*GC*: rs7041 T > G.

### 4.3. Pharmacokinetic Analyses

For each patient, one blood sample was collected immediately before drug intake (Ctrough), under steady-state conditions (5 days after both endovenous (e.v.) and oral (p.o) administration). Plasma samples were obtained by centrifugation at 2000× *g* for 10 min at 4 °C. 6,7-dimethyl-2,3-di(2-pyridyl)quinoxaline (QX) was purchased from Sigma–Aldrich Corporation (Milan, Italy) and used as the internal standard. Analytical standard powders of VRC (purity of 99.97%), PSC (99.83%), and ITC (99.15%) were purchased from Clinisciences (Guidonia Montecelio, Italy). Acetonitrile (HPLC grade) and methanol (HPLC grade) were purchased from VWR (Milan, Italy). Formic acid was from Sigma–Aldrich. HPLC-grade water was produced by a Milli-DI system coupled with a Synergy 185 system by Millipore (Milan, Italy).

VRC, PSC, and ITC quantification were obtained using an HPLC-mass spectrometry system (HLPC-MS), according to a fully validated method [21]. Chromatographic separation was performed at 40 °C, using a column oven, on an Acquity UPLC^®^ BEH C18 (100 mm × 2.1 mm × 1.7 µm) column Waters (Milan, Italy). The mobile phases were composed as follows: phase A, water with formic acid (0.05%); phase B, acetonitrile with formic acid (0.05%). The flow rate was set 1 mL/min, and the total run time was 13 min.

The method was validated following Food and Drug Administration guidelines, and accuracy and precision did not exceed 15%. This work was carried out in a UNI EN ISO 9001:2008 and 13 485:2012 (CE-IVD)-certified laboratory.

### 4.4. Statistical Analyses

The Shapiro–Wilk test was used to test the normality. Non-normal variables were resumed as average ± standard deviation (SD) values, whereas categorical variables were described as numbers and percentages. Kruskal–Wallis and Mann–Whitney tests have been adopted to test differences for continuous variables and genetic groups, considering the level of statistical significance (*p*-value) < 0.05.

Correlations were evaluated through Pearson tests (PC, Pearson coefficient). The predictive power of the considered variables was finally evaluated through univariate (*p* < 0.2) and multivariate (*p* < 0.05) logistic regression analysis.

All the tests were performed with IBM SPSS Statistics 27.0 for Windows (Chicago, IL, USA).

## 5. Conclusions

In conclusion, this is the first study reporting the influence of VD pathway-related gene SNPs on AFU exposures, efficacy, and toxicity. These results could be useful to better understand the mechanism underlying VD actions in the context of AFU metabolism and elimination for tailoring medicine. In fact, genetic analyses coupled with PK are fundamental in order to optimally dose these drugs. Finally, these data could be interesting in order to clarify if the prevalent role of VD is as an immunomodulator; thus, its high concentration could help our immune system or as a drug metabolism and elimination inducer; consequently, its high concentration leads to reduced drug exposure, with a low probability of reaching the clinical outcome.

## Figures and Tables

**Table 1:** Characteristics of subjects involved in the study.

	Voriconazole	Posaconazole	Itraconazole
**No. of patients**	357	136	143
**Caucasians, *n* (%)**	300 (84%)	125 (91.9%)	84 (58.7%)
**Sex (female), *n* (%)**	235 (66.2%)	66 (48.5%)	87 (56.9%)
**Age,** **average ± SD**	42.51 ± 25.01	46.70 ± 19.92	8.78 ± 4.70
**BMI, average ± SD**	21.98 ± 5.29	24.50 ± 4.52	17.13 ± 5.22
**Posology, *n* (%)**	200 BID 184(54.6%) 300 BID 41 (12.2%) 250 BID 14 (4.2%) 150 BID 14 (4.2%) 400 BID 12 (3.6%) 100 BID 10 (3%) others 82 (18.2%)	200 TID 67 (51.5%) 200 BID 23 (17.7%) 400 BID 22 (16.9%) others 24 (13.9)	50 BID 27 (21.8%) 100 BID 23 (18.5%) 200 BID 11 (8.9%) 40 BID 10 (0.1%) others 72 (50.7)
**Administration, *n* (%)**	Oral 192 (53.9%) Endovenous 164 (46.1%)	Oral: 134 (98.5%) Endovenous: 2 (1.5%)	Oral: 137 (95.8%) Endovenous: 6 (4.2%)
**Sepsis, *n* (%)**	8 (2.2%)	0	0
**HIV, *n* (%)**	4 (1.1%)	0	0
**Altered liver function, *n* (%)**	53 (14.9%)	11 (8.1%)	8 (5.6%)
**Altered kidney function, *n* (%)**	13 (3.7%)	8 (5.9%)	5 (3.5%)
**Creatinin, average ± SD**	0.85 ± 0.67	0.76 ± 0.39	0.48 ± 0.47

**Table 2:** Linear regression analysis: factors able to predict voriconazole plasma concentrations over efficacy cut-off limits (>1000 ng/mL) and toxicity cut-off limits (>5000 ng/mL). Bold characters represent statistically significant values.

	Voriconazole Plasma Concentration > 1000 ng/mL				Voriconazole Plasma Concentration > 5000 ng/mL			
	Univariate		Multivariate		Univariate		Multivariate	
	*p*-Value	aOR (95% IC)	*p*-Value	aOR (95% IC)	*p*-Value	aOR (95% IC)	*p*-Value	aOR (95% IC)
**Caucasian**	**0.002**	**2.52 [1.40; 4.5]**	**0.020**	**2.06 [1.12; 3.80]**	**0.118**	**2.037 [0.834; 4.976]**	**0.112**	**2.086 [0.842; 5.465]**
**years > 50 years old**	**0.015**	**1.81 [1.12; 2.92]**			0.519	1.197 [0.693; 2.066]		
**BMI > 25 kg/m^2^**	0.270	1.37 [0.78; 2.41]			0.750	0.902 [0.651; 1.743]		
**Sex (Female)**	**0.185**	**0.72 [0.44; 1.17]**			**0.055**	**0.542 [0.290; 1.014]**	**0.069**	**0.555 [0.294; 1.047]**
***VDR* FokI TC/CC**	**0.087**	**1.70 [0.93; 3.12]**						
***VDR* FokI CC**					**0.101**	**1.580 [0.0915; 2.727]**		
***VDR* Cdx2 AG/GG**	0.407	0.62 [0.21; 1.90]						
***VDR* Cdx2GG**					0.642	0.879 [0.511–1.513]		
***VDR* BsmI GA/AA**	**0.142**	**1.43 [0.89; 2.30]**						
***VDR* BsmI AA**					**0.099**	**1.771 [0.897; 3.496]**		
***VDR* ApaI AA**	0.644	0.89 [0.53; 1.47]			0.957	1.016 [0.562; 1.838]		
***VDR* TaqI CC**	0.898	1.04 [0.56; 1.95]			0.819	0.819 [0.474; 1.415]		
***CYP24A1* 22776 TT**	0.451	1.31 [0.65; 2.60]			0.335	0.659 [0.283; 1.536]		
***CYP24A1* 8620 GG**	0.553	1.18 [0.68; 2.07]			0.406	1.295 [0.704; 2.380]		
***CYP24A1* 3999 CC**	**0.023**	**1.95 [1.10;3.47]**	**0.034**	**1.89 [1.05; 3.40]**				
***CYP24A1* 3999 TC/CC**					**0.102**	**2.010 [0.871; 4.636]**		
***CYP27B1* + 2838 TT**	**0.005**	**1.99 [1.24; 3.22]**	**0.013**	**1.87 [1.14; 3.06]**	0.513	1.189 [0.697; 2.063]		
***CYP27B1* − 1260 TT**	0.601	0.82 [0.39; 1.74]			0.247	1.612 [0.718; 3.618]		
***GC* 1296 AC/CC**	0.352	0.70 [0.42;1.36]			**0.045**	**2.239 [1.019; 4.921]**	**0.037**	**2.349 [1.054; 5.236]**
**Endovenus administration**	0.615	1.13 [0.71; 1.81]			**0.073**	**1.647 [0.955; 2.842]**	**0.033**	**1.833 [1.049; 3.202]**

**Table 3:** Logistic regression analysis with predictors of posaconazole plasma concentrations over the efficacy cut-off limits (>700 ng/mL). Bold characters represent statistically significant values. Bold characters represent statistically significant values. NC: not statistically comparable since one group is missing.

	Posaconazole Plasma Concentrations > 700 ng/mL			
	Univariate		Multivariate	
	*p*-Value	aOR (95% IC)	*p*-Value	aOR (95% IC)
**Caucasian**	**0.193**	**0.435 [0.124; 1.522]**		
**Age > 50 years**	0.577	0.806 [0.378; 1.720]		
**BMI > 25 kg/m^2^**	0.794	1.106 [0.519; 2.354]		
**Sex (Female)**	**0.074**	**0.498 [0.232; 1.070]**		
***VDR*** **FokI CC**	0.750	1.130 [0.533; 2.398]		
***VDR*** **Cdx2 AG/GG**	NC		NC	
***VDR*** **BsmI AA**	0.550	0.697 [0.214; 2.271]		
***VDR*** **ApaI AA**	0.966	0.983 [0.439; 2.201]		
***VDR*** **TaqI CC**	0.281	1.765 [0.629; 4.959]		
***CYP24A1*** **22776 CT/TT**	0.350	0.673 [0.294; 1.542]		
***CYP24A1*** **8620 GG**	**0.147**	**2.170 [0.761; 6.186]**		
***CYP24A1*** **3999 CC**	**0.178**	**1.705 [0.784; 3.709]**		
***CYP27B1*** ** + 2838 CT/TT**	**0.022**	**0.139 [0.026; 0.751]**		
***CYP27B1*** ** − 1260 GT/TT**	**0.022**	**7.197 [1.332; 38.886]**	**0.009**	**15.479 [1.961;122.197]**
***GC*** **1296 CC**	**0.050**	**0.355 [0.126; 1.001]**	**0.036**	**0.286 [0.088; 0.922]**
**Endovenous administration**	NC		NC	

**Table 4:** Logistic regression analysis: factors able to predict itraconazole plasma concentrations over efficacy cut-off limits (>500 ng/mL) (**A**) and toxicity cut-off limits (>1000 ng/mL) (**B**). Bold characters represent statistically significant values. NC: not statistically comparable since one group is missing.

	Itraconazole Plasma Concentrations > 500 ng/mL (A)				Itraconazole Plasma Concentrations > 1000 ng/mL (B)			
	Univariate		Multivariate		Univariate		Multivariate	
	*p*-Value	aOR (95% IC)	*p*-Value	aOR (95% IC)	*p*-Value	aOR (95% IC)	*p*-Value	aOR (95% IC)
**Caucasian**	**0.022**	**0.443 [0.221; 0.889]**			**0.072**	**0.412 [0.157; 1.083]**		
**years > 50 years old**	0.322	1.424 [0.708; 2.866]						
**years < 9 years old**					0.848	1.099 [0.419; 4.179]		
**BMI > 25 kg/m^2^**	0.820	0.865 [2.217; 3.023]			0.561	0.536 [0.065; 4.394]		
**Sex (Female)**	**0.079**	**1.860 [0.930; 3.721]**			**0.006**	**4.469 [1.526; 13.094]**	**0.007**	**4.720 [1.531; 14.552]**
***VDR* FokI CC**	**0.136**	**0.546 [0.246; 1.209]**						
***VDR* FokI TT/CC**					**0.032**	**0.318 [0.112; 0.906]**		
***VDR* Cdx2 GG**	0.873	0.946 [0.476; 1.880]			0.519	0.732 [0.284; 1.888]		
***VDR* BsmI GA/AA**	**0.003**	**0.350 [0.173; 0.707]**	**0.002**	**0.333 [0.164; 0.678]**	**0.041**	**0.357 [0.133; 0.957]**		
***VDR* ApaI CA/AA**	**0.188**	**0.594 [0.273; 1.290]**						
***VDR* ApaI AA**					**0.050**	**2.618 [1.001; 6.847]**		
***VDR* TaqI TC/CC**	0.263	0.675 [0.339; 1.334]			**0.177**	**2.082 [0.717; 6.041]**	**0.034**	**4.050 [1.114; 14.717]**
***CYP24A1* 22776 TT**	0.487	1.359 [0.572; 3.231]			0.691	0.767 [0.207; 2.839]		
***CYP24A1* 8620 AG/GG**	**0.071**	**0.517 [2.552; 1.258]**						
***CYP24A1* 8620 GG**					0.296	0.502 [0.138; 1.826]		
***CYP24A1* 3999 CC**	**0.157**	**1.702 [0.815; 3.555]**			0.947	1.036 [0.369; 2.908]		
***CYP27B1* + 2838 CT/TT**	NC							
***CYP27B1* + 2838 TT**					**0.093**	**2.507 [0.058; 7.239]**		
***CYP27B1* − 1260 GT/TT**	**0.063**	**0.519 [0.260; 1.035]**			**0.022**	**0.304 [0.109; 0.843]**	**0.008**	**0.194 [0.058; 0.650]**
***GC* 1296 AC/CC**	**0.161**	**0.545 [0.234; 1.273]**						
***GC* 1296 CC**					**0.290**	**1.768 [0.615; 6.078]**		
**Endovenus** **administration**	0.984	1.796 [0.349; 9.240]			0.847	1.242 [0.137; 11.221]		

## Data Availability

Data is contained within the article.
